# The “brush sign”: a novel and accessible CT marker for hematoma expansion in hypertensive intracerebral hemorrhage

**DOI:** 10.3389/fneur.2026.1785855

**Published:** 2026-06-01

**Authors:** Jing Wang, Chuyue Wu, Shengli Chen, Qisheng Cheng, Yuxin Ran, Lei He, Zhenjie Yang, Peng Xie, Wangwen Li

**Affiliations:** 1 Affiliated Yongchuan Hospital of Chongqing Medical University, Chongqing, China; 2 Department of Neurology, Chongqing University Three Gorges Hospital, Chongqing, China; 3 Chongqing Key Laboratory of Cerebrovascular Diseases, Chongqing University Three Gorges Hospital, Chongqing, China; 4 Chongqing Municipality Clinical Research Center for Geriatric Diseases, Chongqing University Three Gorges Hospital, Chongqing, China; 5 NHC Key Laboratory of Diagnosis and Treatment on Brain Functional Diseases, The First Affiliated Hospital of Chongqing Medical University, Chongqing, China; 6 Department of Radiology, Chongqing University Three Gorges Hospital, Chongqing, China

**Keywords:** brush sign, computed tomography angiography, hematoma expansion, hypertensive intracerebral hemorrhage, neuroimaging, spot sign

## Abstract

**Objective:**

Patients with hypertensive intracerebral hemorrhage (ICH) are at risk for hematoma expansion (HE) in the early stages. Although the “spot sign” on computed tomography angiography (CTA) is a useful predictor of HE, the method has limitations, including low sensitivity and difficulty in identification. This study introduces a more recognizable and accessible “brush sign” on plain CT images following CTA examination and compares its effectiveness with the “spot sign” in predicting HE.

**Methods:**

Hypertensive ICH patients admitted to the Advanced Stroke Center of the hospital from January 2023 to December 2024 were retrospectively analyzed. This study evaluated sequential plain CT neuroimaging after CTA in these patients, defined the “brush sign,” and identified two morphological types, namely “isolated” and “continuous.” This study analyzed the correlations between the CTA “spot sign,” “brush sign,” and other clinical data with HE. Finally, the HE prediction efficiency of the “brush sign” and “spot sign” was compared using the receiver operating characteristic (ROC) analysis.

**Results:**

A total of 162 hypertensive ICH patients were enrolled, with 45 patients (27.8%) exhibiting HE. The spot, isolated brush, and continuous brush signs were observed in 48 (29.6%), 12 (7.4%), and 43 (26.5%) cases, respectively. The spot sign (*p* = 0.017, OR = 3.943, 95% CI [1.289–12.617]) and continuous brush sign (*p* = 0.016, OR = 3.997, 95% CI [1.302–12.787]) were independent HE predictors. The ROC analysis showed that the continuous brush sign (AUC = 0.895, specificity = 0.949, sensitivity = 0.862) and its combination with the spot sign (AUC = 0.900, specificity = 0.932, sensitivity = 0.852) predicted HE with high accuracy.

**Conclusion:**

The “brush sign” can independently predict early HE in hypertensive ICH patients. The use of the continuous “brush sign” either alone or in combination with the “spot sign” demonstrates high accuracy for predicting HE. These markers can be used to stratify HE risk and suggest early intervention strategies for hypertensive ICH patients.

## 1 Introduction

Intracerebral hemorrhage (ICH) is a subtype of stroke, accounting for 10–15% of cases, second only to ischemic stroke. The ICH incidence is approximately 12–15 per 100,000 people (1). The high morbidity, mortality, and disability rates associated with ICH pose a great burden on the global social economy and medical and healthcare systems (2). According to the Structural vascular lesions, Medication, Amyloid angiopathy, Systemic disease, Hypertension, Undetermined etiological classification system, hypertensive ICH is the most prevalent type of ICH, accounting for approximately 70% of cases (3).

Early hemorrhaging in spontaneous ICH may be persistent and may cause hematoma expansion (HE). HE typically occurs 3–6 h after ICH onset and only rarely after 24 h (4). The incidence of HE varies depending on the site of bleeding (5), and the etiological ICH subtype may also affect the risk of HE (6). HE results from persistent ICH and is the most important factor for deterioration and poor prognosis (4, 5, 7). Therefore, early prediction of HE is crucial for the treatment and rehabilitation of ICH patients.

To identify HE at an early stage, preliminary clinical studies have identified a series of predictive indicators, including physical parameters based on hematoma characteristics such as hematoma volume (8, 9), biomarkers related to vascular injury and inflammation (7), and specific imaging signs. Among the imaging-related parameters, the “spot sign” on computed tomography angiography (CTA) has been proven to have good predictive ability for HE (10, 11). The spot sign has demonstrated accuracy in predicting HE, with a sensitivity of 53% (95% CI, 49–57%) and a specificity of 88% (95% CI, 86–89%), based on the arterial phase, venous phase, and 90-s delay CTA images (12–14). To reduce the adverse effects of secondary injection of contrast agent (15) and the time delay caused by subsequent CT examination (16), a plain CT scan was obtained immediately after the completion of the CTA examination. The range of contrast agent leakage on plain CT scan was then quantified. This study introduced a recognizable and accessible “brush sign” on sequential CT following CTA and compared the outcome with the “spot sign” for predicting HE.

## 2 Methods

### 2.1 Study design

This study was a retrospective analysis of prospectively enrolled participants from an ongoing cohort at the Neurological Department of Chongqing University Three Gorges Hospital. All study protocols were reviewed and approved by the Clinical Trial Ethics Committee of Chongqing University Three Gorges Hospital (Scientific Research No. 20220042). Informed consent was obtained from all subjects or their surrogates according to the Declaration of Helsinki (National Medical Research Registration and archival information System: https://www.medicalresearch.org.cn. Unique identifier: MR-50-23-001346).

### 2.2 Participant recruitment

Patients with spontaneous hypertensive ICH who underwent minimally invasive surgery (MIS) at the Department of Neurology, Chongqing University Three Gorges Hospital between January 2023 and October 2024 were continuously enrolled. The inclusion criteria were as follows (1) age above 18 years; (2) non-pregnant status; (3) diagnosis of spontaneous ICH according to the 2019 Chinese Guidelines for the Diagnosis and Treatment of ICH; (4) no previous episodes of ICH; (5) patiehts who underwent MIS within 24 h of onset; and (6) availability of admission CTA, along with sequential CT, and a follow-up CT to evaluate HE. The exclusion criteria were: (1) secondary cerebral hemorrhage due to tumor, trauma, abnormal vascular structure, and cerebral infarction with hemorrhage transformation; (2) simple ventricular hemorrhage or subarachnoid hemorrhage; (3) undefined hematoma origin or multiple simultaneous ICHs; (4) craniotomy or repeated MIS during hospitalization; and (5) prior history of craniotomy or MIS.

### 2.3 Clinical data

Data were collected from the medical record system, including patients’ demographic data (sex and age), past history (hypertension, diabetes, coronary heart disease, atrial fibrillation, anticoagulant/antiplatelet drug use, previous stroke, smoking status, and alcohol consumption), admission data (height, abdominal girth, weight, blood glucose, oxyhemoglobin saturation, systolic blood pressure, diastolic blood pressure, heart rate, respiratory rate, and body temperature), and admission scores, including the modified Rankin Scale score (mRS) before stroke, mRS after stroke, Glasgow Coma Scale (GCS), and National Institutes of Health Stroke Scale (NIHSS). The CT imaging data included hematoma volume, second CT hematoma volume, time from first CTA/CT to onset, time from second CT to onset, ventricular hemorrhage, subarachnoid hemorrhage, midline shift, and degree of midline shift. The serological indicators included liver injury markers (such as alanine aminotransferase [ALT], aspartate aminotransferase [AST], and *γ*-glutamyl transpeptidase [γ-GT]), indicators related to lipid metabolism (such as cholesterol, triglycerides, high-density lipoprotein [HDL], and low-density lipoprotein [LDL]), and factors associated with coagulation function (such as activated partial thromboplastin time [APTT], prothrombin time [PT], and PT-international normalized ratio [PT-INR]).

### 2.4 Imaging evaluation

On arrival at the emergency department, high-resolution computed tomography angiography (CTA) was acquired per standard protocol with a 256-slice multidetector spiral CT scanner (GE Healthcare, Santa Fe Springs, CA, USA). Image acquisition was performed using a 70 mL contrast agent injector. The scanning parameters included: (1) thickness = 0.625 mm, (2) no slice gap, (3) field of view = 200 mm, (4) matrix size = 512 × 512, and (5) 230–250 mA. Scan coverage extended from the skull base to the apex, and the source images were reformatted into 2.4 mm thick axial, coronal, and sagittal projections. Maximum intensity projection images were usually provided as part of the CTA. Non-contrast plain CT scans were performed immediately after the completion of CTA examination, and the CT scans were reviewed within 24 h using a tandem 16-slice CT scanner.

All CTA images were anonymously and independently reviewed at the same workstation by two experienced readers who were blinded to the patient’s clinical information, outcomes, and other neuroimaging results.

The presence of speckle signs was determined by using previously published methods that show high interrater reliability. The criteria for the spot sign were: ≥1 SPOT, 1–2 mm enhanced lesions, >120 HU, and discontinuity from normal or abnormal vessels (10, 11).

The criteria for identifying the “brush sign” were the appearance of the same spot sign observed on the axial, coronal, and sagittal images of plain CT with an interval of 2.5 mm and with the HU value greater than that of the middle cerebral artery at the same time. The morphological classifications for brush signs were defined as isolated “brush sign” (contrast agent extravasation within 1 mm × 2 mm) or continuous “brush sign”(extravasation >1 mm × 2 mm, showing a linear or curved jet shape) (17).

According to the CT imaging results, hemorrhage sites were categorized as: (1) cerebral lobe hemorrhage, primarily involving the cortex and lower white matter of the cerebral hemisphere; (2) deep hemorrhage, primarily involving the basal ganglia, the ventricular white matter, the thalamus, or the internal capsule; (3) cerebellar hemorrhage, including the cerebellar cortex and deep cerebellum; and (4) brainstem hemorrhage, involving the pons (18).

The clot size was calculated in mL using the ellipsoid formula (A × B × C/2). All patients underwent plain CT scanning within 24 h of onset, and the images were reviewed. Absolute and relative percentage changes in hematoma size (mL) were calculated to assess HE. The main outcome of this study was significant HE, defined as either a ≥ 12.5 mL increase in absolute hematoma size or a ≥ 33% relative increase.

### 2.5 Statistical analysis

The χ^2^ test was used to test for differences in categorical variables. The Kolmogorov–Smirnov test was used to evaluate the normality of continuous variables. The student’s *t*-test was used for independent samples, and the Mann–Whitney *U* test was used for non-normally distributed data. Data are expressed as the mean ± standard deviation, median (interquartile range [IQR]), or number (percentage). First, we aimed to assess the associations between the “spot sign,” “brush sign,” “continuous brush sign,” and/or “isolated brush sign” with HE. Then, we calculated the other factors associated with HE. To further assess independent risk factors for clinical outcome, binary and multivariate logistic regression analyses were performed. We drew ROC curves for the prediction of HE with either individual CTA signs or combinations of CTA signs and calculated the AUC and *p* values of the curves. The sample size was estimated to be 155–180 based on previous similar studies of CTA signs and ICH (1, 8, 12), along with the number of predictor variables included in the regression models. This sufficient sample size ensured that it met the requirements of multivariate statistical analysis, guaranteed the stability of parameter estimation, and supported the reliability of the study conclusions. SPSS 22.0 was used as a statistical tool for all statistical analyses, and a *p*-value of < 0.05 was considered statistically significant.

## 3 Results

### 3.1 Baseline characteristics of participants

**Figure 1:**
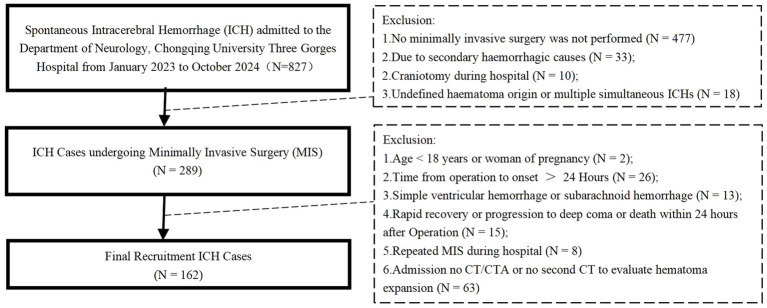
Flow diagram of eligibility cases of spontaneous hypertensive intracerebral hemorrhage (ICH).

A flowchart outlining the eligibility of ICH cases is shown in Figure 1. A total of 162 ICH patients after MIS were included, comprising 96 male patients and 66 female patients. The mean age was 63.73 ± 11.61 years, with a median age of 65 (interquartile range: 56–74). Table 1 shows the baseline data of these patients.

**Table 1:** Baseline characteristics of participants (*n* = 162).

Characteristics	Data
Sex, male	96 (59.3%)
Age, years	63.73 ± 11.61
Hematoma expansion	45 (27.8%)
Location of bleeding	
Lobar hemorrhage	16 (9.9%)
Deep hemorrhage	138 (85.2%)
Cerebellar hemorrhage	6 (3.7%)
Brainstem hemorrhage	2 (1.2%)
Bleeding types	
Ventricular hemorrhage	80(49.4%)
Subarachnoid hemorrhage	15 (9.3%)
Midline displacement	115 (71.0%)
The degree of midline displacement	7.53 ± 3.73 mm
Medical history	
Hypertension	107 (66.0%)
Diabetes	9 (5.6%)
Ischemic heart disease	10 (6.2%)
Atrial fibrillation	2 (1.2%)
Anticoagulant/antiplatelet drug use	4 (2.5%)
Stroke	19 (11.7%)
Smoking status	56 (34.6%)
Alcohol consumption	58 (35.8%)
Image factors	
Hematoma volume evaluated in sequential CT after CTA, mL	29.92 ± 20.50
Hematoma volume of the second CT, mL	36.09 ± 27.61
Time from the onset of the CTA examination, hours	4.56 ± 4.08
Time from the sequential CT examination, hours	4.73 ± 4.29
Time from the second CT, hours	11.07 ± 6.81
Scores	
mRS Score before ICH	0 (IQR = 0)
mRS Score after ICH	5 (IQR = 1)
Admission GCS score	10 (IQR = 7)
Admission NIHSS score	16 (IQR = 8)

The majority of ICH cases were classified as deep hemorrhage (138/85.2%). There were 80 cases of ventricular hemorrhage (49.4%) and 115 cases of midline displacement (71.0%) with a displacement degree of 7.53 ± 3.73 mm. A total of 107 (66.0%) patients had a history of hypertension. There were 56 cases (34.6%) of smoking and 58 cases (35.8%) of alcohol consumption. A total of 45 patients (27.8%) exhibited HE, with 29 (64.4%) male patients and 16 (35.6%) female patients at a median age of 67 (IQR 56–72) years.

### 3.2 Correlation between CTA “spot sign” or sequential CT signs and HE

**Figure 2:**
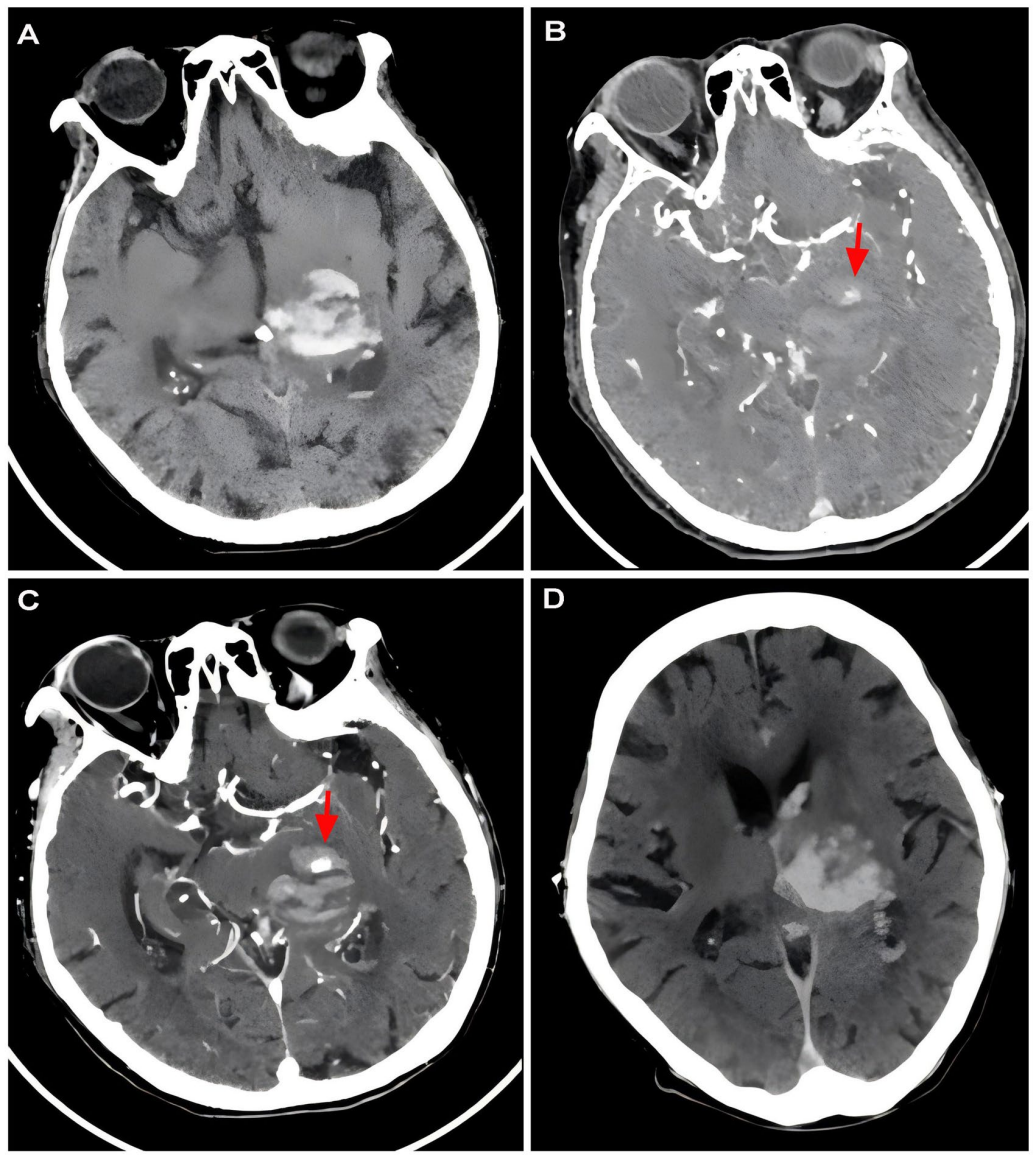
A 75-year-old woman with a left thalamic hemorrhage. The onset time was 13:00 on 30 April, and the time of arrival at the emergency department of the hospital was 14:20. **(A,B)** First CT and enhanced CT at 14:36, a hematoma volume was 12.90 mL, spot sign positive (red arrow); **(C)** sequential CT scan at 14:40 with brush sign positive and isolated morphology (red arrow); and **(D)** re-examined CT at 18:24, and the hematoma volume was 13.55 mL without significant hematoma expansion.

**Figure 3:**
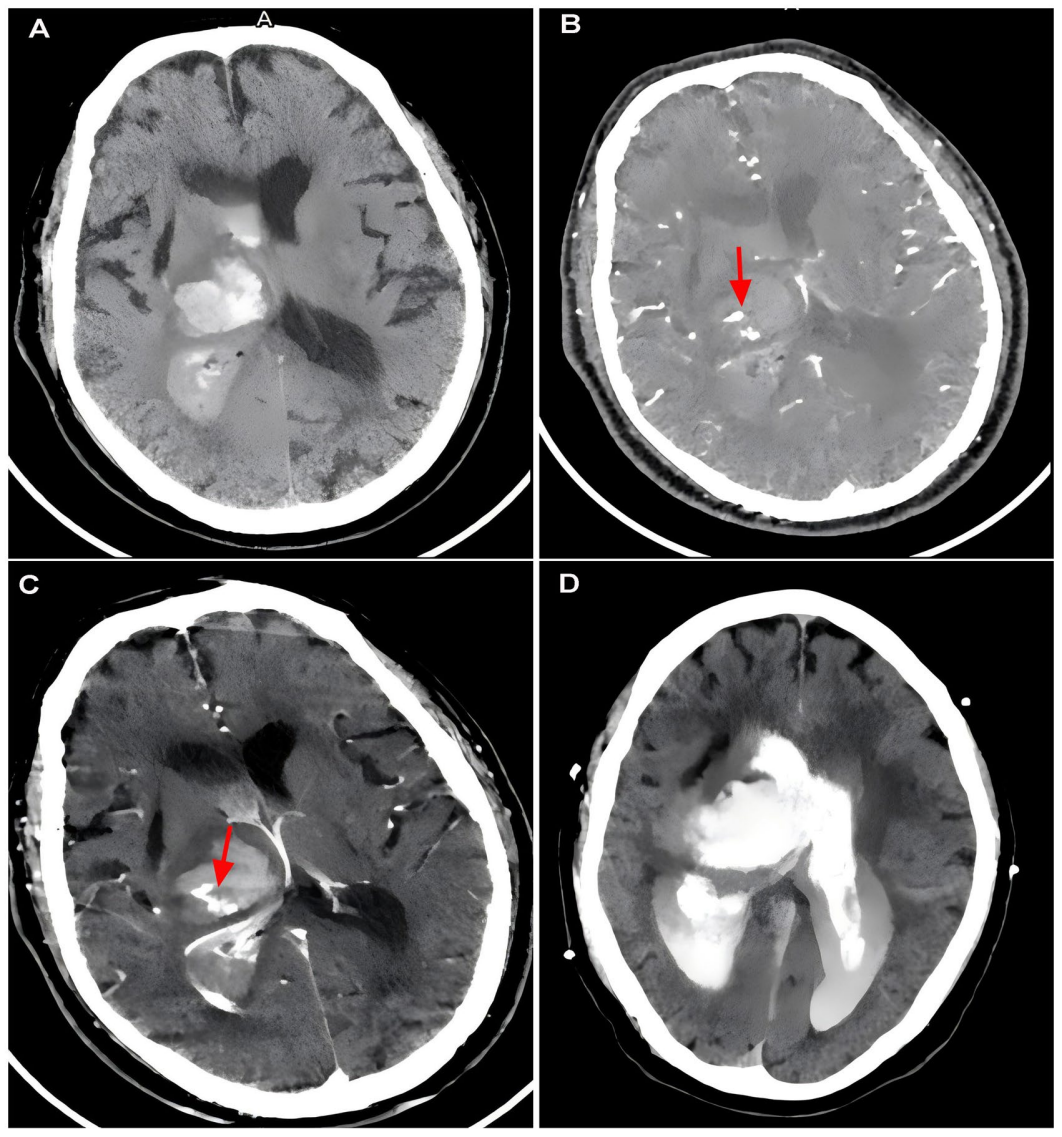
A 79-year-old man with hemorrhage in the right basal ganglia, thalamic-lateral ventricle. The onset time was 4:16 on 10 July, and the time of arrival at the emergency department of our hospital was 6:00. **(A,B)** first CT and enhanced CT at 6:12, hematoma volume was 9.45 mL, spot sign positive (red arrow); **(C)** sequential CT scan at 6:16 with positive brush sign and continuous morphology (red arrow); and **(D)** re-examined CT at 9:53, and the hematoma volume was 20.20 mL, which showed obvious hematoma expansion.

**Figure 4:**
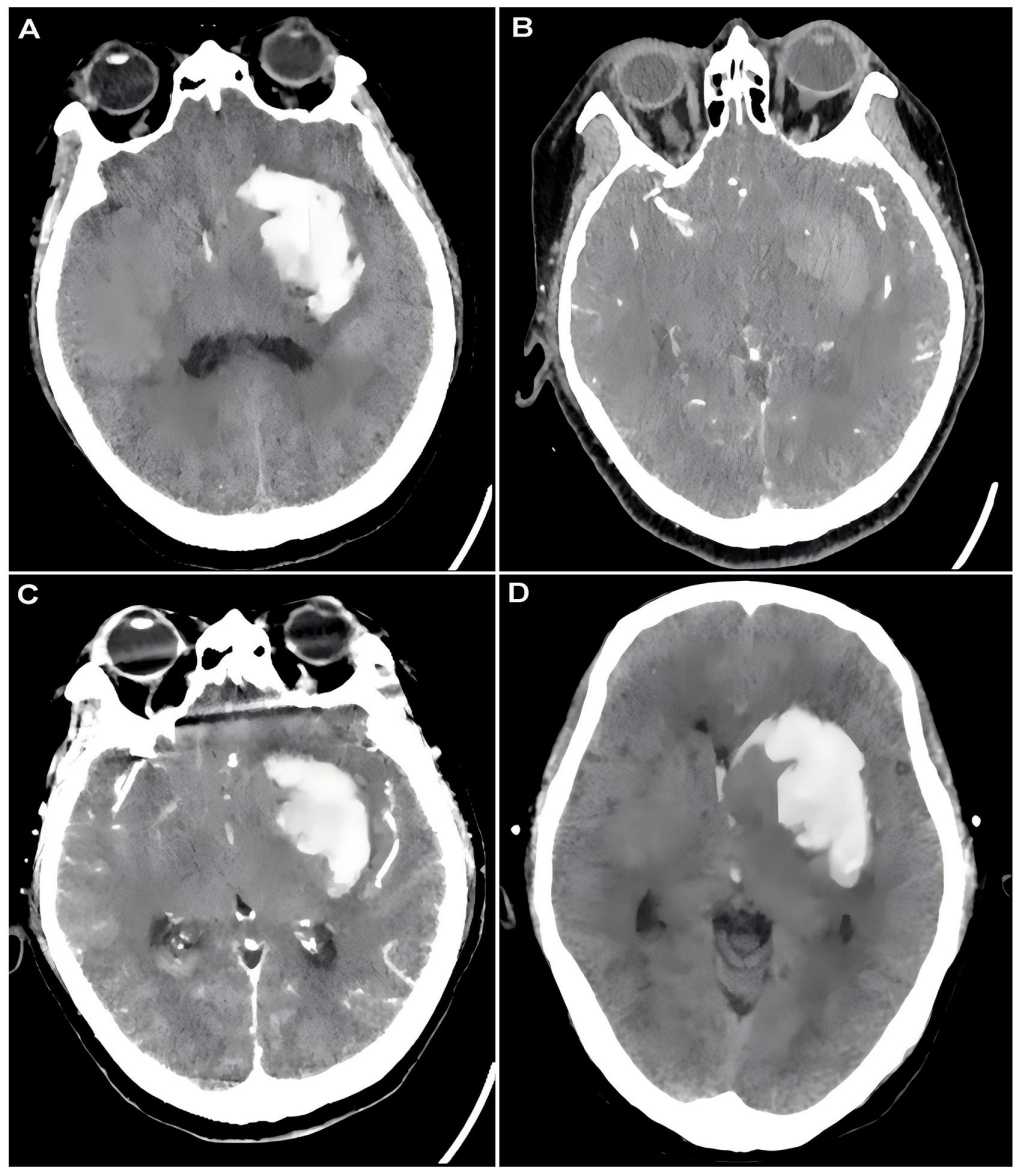
A 37-year-old woman with hemorrhage in the left basal ganglia, external capsule, insula, paraventricular, and frontal lobes. The onset time was 7:26 on 17 January, and the time of arrival at the emergency department of our hospital was 10:36. **(A,B)** First CT and enhanced CT at 10:55, hematoma volume was 27.48 mL, spot sign was negative; **(C)** sequential CT scan at 10:56 with brush positive and isolated morphology; and **(D)** re-examined CT at 14:59, the hematoma volume was 29.07 mL without significant hematoma expansion.

**Figure 5:**
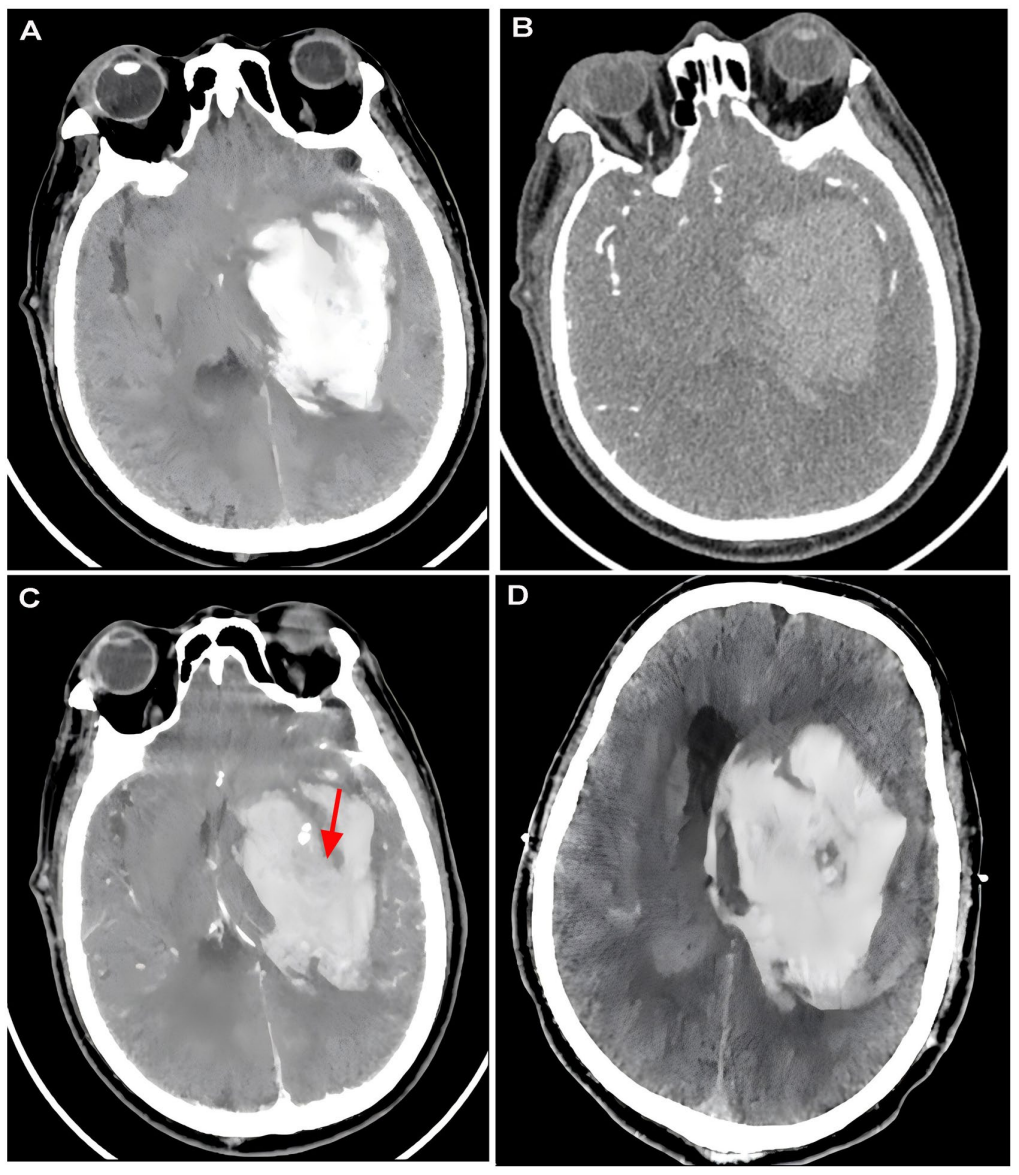
A 77-year-old man with hemorrhage in the left basal ganglia and frontal parietal lobe. The onset time was 1:26 on 20 September, and the time of arrival at the emergency department of our hospital was 8:36. **(A,B)** First CT and enhanced CT were performed at 8:50. The hematoma volume was 118.50 mL, and the spot sign was negative. **(C)** Sequential CT scan at 8:52, brush sign was positive, and morphology was continuous (red arrow); and **(D)** re-examined CT at 11:47, the hematoma volume was 160.00 mL, which showed obvious hematoma expansion.

Figures 2–5 show four ICH cases, each demonstrating different statuses of the “brush sign” and “spot sign.” For single signs, “spot sign” was observed on CTA in 48 cases (29.6%). The “brush sign” was observed in 55 cases (34.0%) on sequential CT after CTA, among which 43 (26.6%) were continuous and 12 (7.4%) were isolated. For combinatorial signs, 42 cases (25.9%) showed both spot and brush signs, 38 cases (23.5%) showed spot and continuous brush sign, 4 cases (2.5%) showed spot and isolated brush signs, 61 cases (37.7%) showed either spot or brush signs, 53 cases (32.7%) showed either spot or continuous brush signs, and 56 cases (34.6%) showed either spot sign or isolated brush signs. The correlation between CTA “spot sign” or sequential CT signs with HE, for both single signs and combinatorial signs, is shown in Table 2.

**Table 2:** Correlation between CTA “spot sign” or sequential CT signs and HE for single signs and combinatorial signs.

Imaging signs	Cases (*N* = 162) *N*	HE (*N* = 45) *N*(%)	Non-HE (*N* = 117) *N*(%)	*P*	OR	95% CI
Spot sign	48	32(71.1)	16(13.7)	**<0.001**	15.538	6.756~35.737
Brush sign	55	37(82.2)	18(15.4)	**<0.001**	25.437	10.194~63.477
Continuous	43	36(80.0)	7(6.0)	**<0.001**	62.857	21.843~180.885
Isolated	12	1(2.2)	11(9.4)	0.152	0.219	0.027~1.748
Spot and brush signs	42	32(71.1)	10(8.5)	**<0.001**	26.338	10.558~65.702
Spot and continuous brush signs	38	32(71.1)	6(5.1)	**<0.001**	45.538	16.028~129.383
Spot and isolated brush signs	4	0(0.0)	4(3.4)	0.999	11.239	5.036~25.082
Spot or brush signs	61	37(82.2)	24(20.5)	**<0.001**	17.922	7.388~43.477
Spot or continuous brush signs	53	36(80.0)	17(14.5)	**<0.001**	23.529	9.631~57.484
Spot or isolated brush signs	56	33(73.3)	23(19.7)	**<0.001**	11.239	5.036~25.082

The bold font indicates *p* < 0.05.

For single signs, more “spot sign,” “brush sign,” and more “continuous brush sign” were observed in the 45 HE cases than in the 117 non-HE cases (*p* < 0.001). For combinatorial signs, more “spot and brush signs,” “spot and continuous brush signs,” “spot or brush signs,” “spot or continuous brush signs,” and more “spot or isolated brush signs” were observed in the HE patients than in non-HE patients (*p* < 0.001).

### 3.3 Analysis of influencing factors of HE

Demographic data of ICH patients with and without HE are shown in Table 3. The univariate analysis showed that HE was associated with hematoma volume evaluated in sequential CT after CTA, second CT hematoma volume, time from CTA to onset, time from sequential CT to onset, time from second CT to onset, ventricular hemorrhage, and degree of midline shift (*p* < 0.05).

**Table 3:** Demographic data of intracerebral hemorrhage patients with and without hematoma expansion (HE).

Characteristic data	Variables		HE (*N* = 45) N(%)/Mean ± SD/Median (IQR)	Non-HE (*N* = 117) N(%)/Mean ± SD/Median (IQR)	*P*	OR	95% CI
Demographic	Age, years		64.98 ± 10.87	63.26 ± 11.90	0.398	1.013	0.983~1.044
	Sex, male		29(64.44)	67(57.26)	0.406	0.739	0.363~1.507
Past history	Hypertension		28(62.22)	79(67.52)	0.524	0.792	0.387~1.621
	Diabetes		3(6.67)	6(5.13)	0.703	1.321	0.316~5.526
	Coronary heart disease		5(11.11)	5(4.27)	0.118	2.800	0.77~10.183
	Atrial fibrillation		1(2.22)	1(0.85)	0.496	2.636	0.161~43.071
	Anticoagulant/antiplatelet drug use		4(8.89)	0(0.00)	0.999	/	/
	Previous stroke		7(15.56)	12(10.26)	0.351	1.612	0.591~4.396
	Smoking status		19(42.22)	37(31.62)	0.206	1.580	0.778~3.209
	Alcohol consumption		19(42.22)	39(33.33)	0.292	1.462	0.722~2.959
Admission data	Random Blood glucose, mmol/L		7.00(2.93)	7.10(2.30)	0.862	1.013	0.873~1.177
	Fasting blood glucose, mmol/L		7.12(2.24)	7.19(2.55)	0.599	0.962	0.831~1.113
	Glycosylated hemoglobin		5.89(0.43)	5.89(0.36)	0.945	0.988	0.705~1.386
	Systolic blood pressure, mmHg		172.50(36.75)	170.00(33.50)	0.817	1.002	0.989~1.014
	Diastolic blood pressure, mmHg		96.00(26.75)	95.00(26.50)	0.897	0.999	0.982~1.016
Admission score	mRS before ICH		0(0)	0(0)	0.272	1.330	0.799~2.214
	mRS after ICH		5(1)	5(1)	0.502	0.844	0.513~1.387
	GCS		10(7)	11(7)	0.709	0.982	0.892~1.081
	NHISS		17(9.50)	16(7)	0.386	1.024	0.971~1.079
CT imaging data	Hematoma location	Lobar	3(6.7)	13(11.1)	0.863	0.849	0.771~0.929
		Deep	41(91.1)	97(82.9)			
		Cerebellar	0(0.0)	6(5.1)			
		Brainstem	1(2.2)	1(0.9)			
	Hematoma volume, mL		28.71 ± 26.79	30.38 ± 17.62	**0.033**	2.133	1.082~3.826
	Second CT hematoma volume, mL		50.78 ± 41.00	30.44 ± 17.46	**<0.001**	1.028	1.013~1.044
	Time from CTA to onset, h		2.83 ± 2.00	5.22 ± 4.48	**0.003**	0.710	0.609~0.932
	Time from sequential CT to onset, h		3.07 ± 2.73	5.36 ± 4.61	**0.002**	0.793	0.686~0.918
	Time from second CT to onset, h		9.05 ± 5.95	11.85 ± 6.98	**0.023**	0.928	0.870~0.990
	Ventricular hemorrhage		29(64.44)	51(43.59)	**0.019**	2.346	1.152~4.778
	Subarachnoid hemorrhage		5(11.11)	10(8.55)	0.615	1.337	0.431~4.154
	Midline shift		36(80)	79(67.52)	0.121	1.924	0.842~4.397
	Degree of midline shift, mm		9.47 ± 3.93	6.64 ± 3.30	**<0.001**	1.239	1.099~1.397
Serological indicators	Alanine Aminotransferase, ALT (U/L)		18.6(12.25~30.62)	16.8(12.425~25.925)	**0.024**	1.031	1.004~1.059
	Aspartate Aminotransferase, AST (U/L)		27.2(20.45~33.75)	24.85(20.05~31.675)	0.450	1.009	0.986~1.033
	γ-glutamyl transpeptidase, γ-GT (U/L)		25(17.5~29)	18.5(13~33.75)	0.840	1.000	0.996~1.005
	Cholesterol, mmol/L		4.39(3.495~4.39)	4.39(4.145~4.54)	**0.016**	0.534	0.320~0.890
	Triglycerides, mmol/L		1.59(1.065~1.59)	1.59(0.95~1.59)	0.657	0.929	0.672~1.284
	High density lipoprotein, HDL, mmol/L		1.4(1.18~1.46)	1.4(1.2525~1.4325)	0.680	0.814	0.305~2.170
	Low-density lipoprotein, LDL, mmol/L		2.67(1.99~2.67)	2.67(2.455~2.9275)	**0.011**	0.470	0.262~0.840
	Activated Partial Thromboplastin Time, APTT, s		24.9(23.4~27.2)	24.1(22.35~25.825)	**0.008**	1.169	1.041~1.312
	Prothrombin time, PT, s		10.8(10.3~11.05)	10.8(10.3~11.3)	0.277	1.137	0.902~1.432
	PT-international normalized ratio, PT-INR		0.94(0.915~0.975)	0.94(0.89~0.99)	0.295	2.360	0.473~11.783

The bold font indicates *p* < 0.05.

The multi-factor analysis (Table 4) showed that HE was associated with spot sign (*p* = 0.017), continuous brush sign (*p* = 0.016), hematoma volume (*p* = 0.033), time from sequential CT to onset (*p* = 0.040), alanine aminotransferase (*p* = 0.028), low-density lipoprotein (*p* = 0.026), and activated partial thromboplastin time (*p* = 0.028).

**Table 4:** Multivariate regression analysis of hematoma expansion (HE).

Indicator	VIF*	To.	*P*	OR	95%CI	
					Lower	Upper
Spot sign	1.25	0.80	0.017	3.943	1.289	12.617
Continuous brush sign	1.31	0.76	0.016	3.997	1.302	12.787
Hematoma volume, mL	1.42	0.70	0.033	1.028	1.012	1.050
CT time ^, h	1.28	0.78	0.040	0.813	0.641	0.987
ALT (U/L)	1.19	0.84	0.028	1.043	1.006	1.080
LDL, mmol/L	1.15	0.87	0.026	0.399	0.170	0.870
APTT, s	1.22	0.82	0.028	1.216	1.034	1.469

* This study conducted a preliminary assessment of collinearity for the included independent variables (using the VIF analysis, with VIF < 5: there is no significant multicollinearity among the variables, and they can be included in the regression model). The results showed that the VIF values of all the independent variables included in the multivariate regression model in this study were all < 3, far below the critical value of 5, suggesting that there is no significant multicollinearity among the variables, and meeting the applicable conditions for multivariate logistic regression analysis.

^ CT time: Time from sequential CT to onset; VIF, Variance inflation factor; To., Tolerance; ALT, Alanine aminotransferase, LD, Low-density lipoprotein; APTT, Activated partial thromboplastin time.

### 3.4 CTA “spot sign” or sequential CT signs to predict HE

**Figure 6:**
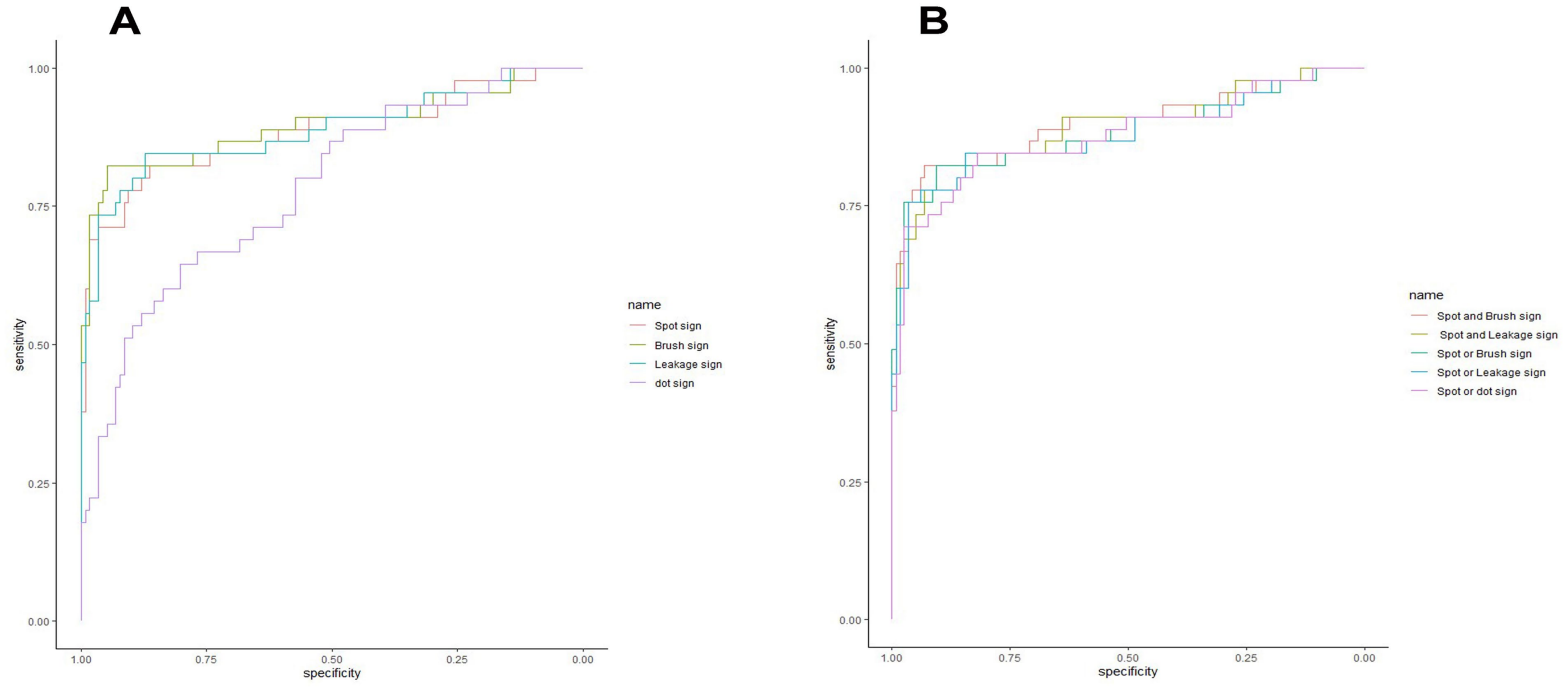
The ROC curves of CTA “spot sign” or sequential CT signs to predict hematoma expansion (HE) for single signs. **(A)** Single signs and **(B)** Combinatorial signs.

The ROC curves of CTA “spot sign” or sequential CT signs for predicting HE are shown in Figure 6. The AUC values, specificity values, and sensitivity values are shown in Table 5. For single signs, the continuous brush sign had the highest AUC value of 0.895, with the highest specificity of 0.949, and the highest sensitivity of 0.862. For combinatorial signs, the spot and continuous brush signs had the highest AUC value of 0.900, the highest specificity of 0.932, and the highest sensitivity of 0.852. There were no HE cases with the spot sign combined with the isolated brush sign. For predicting the efficacy of HE, the AUC value, sensitivity, and specificity of the combinatorial signs were generally better than those of the single signs.

**Table 5:** The AUC values, specificity values, and sensitivity values of CTA “spot sign” or sequential CT signs to predict hematoma expansion (HE).

Prediction method	Imaging signs	AUC	Specificity	Sensitivity	*P*
Single signs	Spot sign	0.883	0.863	0.822	0.186
	Brush sign	0.887	0.872	0.844	0.348
	Continuous	0.895	0.949	0.862	comparison object
	Isolated	0.780	0.803	0.644	**<0.001**
Combinatorial signs	Spot and brush signs	0.892	0.906	0.822	0.169
	Spot and continuous brush signs	0.900	0.932	0.852	comparison object
	Spot and isolated brush signs	/	/	/	/
	Spot or brush signs	0.880	0.996	0.756	0.102
	Spot or continuous brush signs	0.885	0.974	0.756	0.145
	Spot or isolated brush signs	0.877	0.974	0.711	**0.048**

The bold font indicates *p* < 0.05.

## 4 Discussion

Previous studies have reported that the HE incidence is approximately 20–30% (19–22). This study reported an HE incidence rate of 27.8% (45/162), which is consistent with previous studies (19–22). The “spot sign” was observed in 48 cases (29.6%), basically consistent with the incidence rates reported in previous studies (21–30%) (10–13). This study found that the spot sign and continuous brush sign on sequential CT after CTA were independent predictors of HE. In addition, hematoma volume, time from sequential CT to onset, alanine aminotransferase, low-density lipoprotein, activated partial thromboplastin time, and APTT were also independent risk factors for HE. Among the individual signs, the continuous brush sign had the highest AUC value of 0.895, with the highest specificity of 0.949 and the highest sensitivity of 0.862. For combinations of signs, the spot and continuous brush signs together had the highest AUC value of 0.900, with a specificity of 0.932 and the highest sensitivity of 0.852, compared with other sign combinations.

Another predictor of HE is the time between the first CT scan and onset. The shorter the time, the higher the probability of observing HE in early CT review, and vice versa (23, 24). The reason may be that hematomas are not completely stable in the early stage, and the mass continues to bleed slowly during the CT examination. Hematoma volume was also identified as a predictor. Broderick (8) found that the incidence of early HE in patients with an initial hematoma volume below 20 mL was much lower than in patients with an initial hematoma volume above 20 mL. Weimar (9) found that, compared with patients with an initial hematoma volume above 30 mL, patients with a hematoma volume below 20 mL had a significantly lower probability of HE occurrence. One possible reason is that the bleeding itself is difficult to stop due to the rupture of large blood vessels associated with a larger hematoma volume. Second, patients with a large hematoma volume also exhibit slow bleeding of vessels around the hematoma, which accelerates HE (20, 25). The study results also showed that the time of onset of the first CT and the initial hematoma volume were independent risk factors for HE, which is consistent with the conclusions of previous studies (8, 9, 20, 23–25). In addition, these results also showed that some abnormal biochemical indicators, as reported in previous studies, such as alanine aminotransferase, representing liver function (26, 27), low-density lipoprotein, reflecting lipid metabolism (28, 29), and APTT, reflecting coagulation function (30, 31), were also associated with HE.

In HE cases, clinical influencing factors are often too heterogeneous and not intuitive to interpret, or their evaluation takes a long time; these delays may lead to patients who need surgery missing the optimal treatment window. With the development of imaging technology, specific imaging signs have been used for the prediction and assessment of HE. In 2007, Wada et al. (32) first proposed the CTA spot sign, which has been continuously developed into the current widely accepted definition, consisting of the following: (1) at least one area of contrast agent concentration appears at the extravasation site within the intracranial hematoma; (2) compared with the density of surrounding hematoma, the CT measurement value is greater than 120 HU; (3) adjacent normal or abnormal blood vessels run discontinuously around the hematoma; and (4) spot signs exist within the range of intracranial hematoma (33–35). The spot sign has proven to be an important factor in determining the enlargement of hematoma expansion (33–35). Based on this mechanism, many scholars have proposed improved spot signs, such as those identified on delayed CTA, venous CTA, dynamic CTA, and CT perfusion (CTA) (12–14, 36, 37). The spot sign has proven to be an attractive tool for therapeutic interventions in ICH patients to prevent or reduce HE (38). However, due to the low sensitivity of the spot signs in predicting HE, Kimihiko, a Japanese scholar, proposed a new marker, the “leakage sign,” for predicting HE in 2016 (39). Patients were required to undergo two CTA scans, one in the CTA phase and the other in the delayed phase (5 min after the CTA phase). A region of interest (ROI) with a diameter of 10 mm was set, and its CT value was calculated. The phenomenon in which the CT value in the ROI in the delayed phase increased by >10% compared with that in the CTA phase was defined as a leakage sign. Some studies have shown that the leakage sign has higher sensitivity (93.3%) and specificity (88.9%) than the spot sign in predicting HE. However, the methods for obtaining leakage sign images have drawbacks. First, CTA scanning requires contrast agent administration, which is time-consuming. In addition, repeated contrast injections carry an increased risk of physical adverse effects and injury (15). In addition, the frequency and predictive value of CTA spot signs decrease with the time from symptom onset to CTA, and the frequency of late CTA spots decreases by 2/3 compared with early CTA spots (0–2 h) (16). Furthermore, this study found that spot sign recognition on CTA was difficult because the spot sign did not stand out from the background, but the contrast agent leakage on the plain CT scan was much easier to identify, even without professional physician training, and could still be accurately identified.

Therefore, a better approach to improve the accuracy of HE prediction involves combining spot signs and other imaging signs (such as the blend sign and the Black-and-White sign) and finding the best intervention for the population, as reported in recent studies (40, 41). The ROC curve analysis in this study showed that the single continuous brush sign (AUC = 0.895, specificity = 0.949, and sensitivity = 0.862) and the combination of the spot and continuous brush signs (AUC = 0.900, specificity = 0.932, sensitivity = 0.852) had the highest performance in predicting HE. In addition, this study found that the AUC value, sensitivity, and specificity of the combinatorial signs were generally superior to those of the single signs for predicting HE.

In a 2018 study, Vedicherla et al. (17) subdivided spot signs into two different forms, namely: (1) the “dot sign,” defined as a 1 mm × 2 mm contrast exosmosis point, and (2) the “blush sign,” defined as being greater than 1 mm × 2 mm and having a curved jet appearance. The “blush sign” was found to be an independent predictor of HE in primary hypertensive hemorrhage. This study referred to the definition of the above forms and described and defined two forms of “brush sign,” namely, the “continuous” and “isolated” brush signs, on sequential CT after CTA. Koculym et al. (42) showed that the sensitivity of the “spot sign” to HE depends on the leakage rate of the contrast agent. A linear or curvilinear jet of contrast agent leakage was found to be associated with higher bleeding rates in the gastrointestinal tract (42). Similarly, in hypertensive ICH, the shape on the image, namely, the shape formed by the “continuous brush sign” according to this study, may better reflect the rapid bleeding accumulation from blood vessels under the continuous pressure of hypertension (43). The other form, namely, the “isolated brush sign” as defined, represents only a localized sign of contrast agent leakage that we have identified and may also be a manifestation of other pathological changes, such as pseudoaneurysm (34). The biological basis for the morphological variability of the CTA “spot sign” remains unclear. The mechanism underlying these findings may be that, in patients with underlying vascular disease (such as hypertensive hyaline), the primary source of bleeding is ruptured diseased blood vessels, which cause secondary bleeding that spreads and destroys surrounding arteries due to the mass effect. This study also shows that continuous contrast agent leakage on sequential CT plain scans after CTA is more sensitive than isolated contrast agent leakage. These findings are consistent with a mechanism of enlargement of hematoma caused by the rupture or collapse of multiple blood vessels. Isolated contrast agent leakage suggests a lower risk of rebleeding, and the body can stop the bleeding through its own coagulation mechanism. When a sequential plain CT scan after CTA can replace a second CTA examination, patient waiting time does not increase, while the intake of contrast media and costs decrease, which is beneficial for clinical implementation.

It should be noted that a notable advantage of these radiomics-based workflows is that they do not require additional imaging acquisition, thereby avoiding the increase in radiation exposure associated with supplementary scans. This provides an alternative approach for cerebral edema prediction in clinical practice, and the findings of this study can be viewed as a complementary strategy, particularly in scenarios where immediate evaluation of new-onset deficits necessitates additional non-contrast CT scans.

Furthermore, this study used the classic ABC/2 method for hematoma volume measurement, which is simple and is widely used in clinical practice (44). Compared with semi-automatic methods, the ABC/2 method is more accurate but more time-consuming; compared with automatic methods, the ABC/2 method is more efficient but more dependent on the image quality. The uniform use of the ABC/2 method ensures internal validity, though this method may introduce measurement differences, as specified in previous studies (45, 46).

This study has several limitations. First, this study was a retrospective observational study, which was inherently susceptible to selection bias and information bias. These potential biases may limit the generalizability of the findings. To minimize these biases, this study adopted strict and consistent inclusion and exclusion criteria, enrolled consecutive eligible patients, invited independent blinded reviewers to interpret imaging findings, and adjusted for potential confounders using multivariate logistic regression analysis. Second, the retrospective study involved a specific population from a single center and lacked follow-up imaging, which may have led to the underestimation or overestimation of the predictive value of this imaging sign for hematoma enlargement. Third, the study included many patients who were imaged within 6 h of symptom onset (mean 4.56 ± 4.08 h for first CTA), which may have resulted in smaller baseline ICH volumes and fewer visible “spot sign” features. Fourth, although an additional CT scan increases radiation exposure, sequential plain CT after CTA enhancement improves standardization, enhances operability, and reduces patient waiting time during CT, particularly in coma patients. The longer the waiting time, the greater the risk. Achieving the same prediction effect for hematoma enlargement by delaying CT acquisition for several minutes and obtaining a CT plain scan instead of an additional CTA scan can reduce contrast exposure, lower the risk of renal function damage, reduce costs, and improve clinical feasibility. Finally, the interpretation of the results measured using the ABC/2 method may introduce bias, as different measurement methods may have varying degrees of deviation.

## 5 Conclusion

The continuous brush sign observed in sequential CT following CTA is based on imaging biomarkers such as the “spot sign.” Prediction of HE using the continuous brush sign, alone or in combination with the spot sign, demonstrates high accuracy and can serve as a risk stratification indicator for early intervention in hypertensive ICH patients. The improved sensitivity of the continuous brush sign, alone or in combination with the spot sign in predicting HE, may enhance patient selection for hemostatic therapy.

## Data Availability

The raw data supporting the conclusions of this article will be made available by the authors, without undue reservation.
